# TIME BY YOURSELF: THEORETICAL BACKGROUND AND FINDINGS FROM THE NEW POSITIVE SOLITUDE QUESTIONNAIRE

**DOI:** 10.1093/geroni/igad104.1056

**Published:** 2023-12-21

**Authors:** Yuval Palgi, Dikla Segel-Karpas, Sharon Ost Mor, Yaakov Hoffman, Ehud Bodner

**Affiliations:** University of Haifa, Haifa, Hefa, Israel; University of Haifa, Haifa, Hefa, Israel; University of Haifa, Haifa, Hefa, Israel; Bar Ilan University, Ramat Gan, HaMerkaz, Israel; Bar Ilan University, Tel Aviv, Tel Aviv, Israel

## Abstract

The present study describes the development of a new questionnaire for measuring positive solitude. First we will discuss the conceptualization of positive solitude, and refer to the contradictions that exist in the literature regarding this concept. We will then present the theoretical background on which we based our questionnaire, and some empirical findings regarding its utility. We will describe the 9-item tool and its relationships to positive and negative measures of well-being and mental health. Furthermore, we will discuss findings from a Holocaust survivors study showing the role of positive solitude on posttraumatic symptoms. We will conclude this presentation by discussing several future directives, some of which that will be discussed in other presentations of this symposium.

